# A diagnostic model for serious COVID-19 infection among older adults in Shanghai during the Omicron wave

**DOI:** 10.3389/fmed.2022.1018516

**Published:** 2022-12-19

**Authors:** Suxia Bao, Guanzhu Lu, Yaoyue Kang, Yuanyuan Zhou, Yuhuan Wang, Lei Yan, Donglin Yin, Yujie Bao, Xiaoling Yuan, Jie Xu

**Affiliations:** Department of Infectious Disease, Shanghai Ninth People’s Hospital, Shanghai Jiao Tong University School of Medicine, Shanghai, China

**Keywords:** diagnose model, discriminate, serious COVID-19 infection, geriatric patients, Shanghai diagnose model, Shanghai

## Abstract

**Background:**

The Omicron variant is characterized by striking infectivity and antibody evasion. The analysis of Omicron variant BA.2 infection risk factors is limited among geriatric individuals and understanding these risk factors would promote improvement in the public health system and reduction in mortality. Therefore, our research investigated BA.2 infection risk factors for discriminating severe/critical from mild/moderate geriatric patients.

**Methods:**

Baseline characteristics of enrolled geriatric patients (aged over 60 years) with Omicron infections were analyzed. A logistic regression analysis was conducted to evaluate factors correlated with severe/critical patients. A receiver operating characteristic (ROC) curve was constructed for predicting variables to discriminate mild/moderate patients from severe/critical patients.

**Results:**

A total of 595 geriatric patients older than 60 years were enrolled in this study. Lymphocyte subset levels were significantly decreased, and white blood cells (WBCs) and D-dimer levels were significantly increased with disease progression from a mild/moderate state to a severe/critical state. Univariate and multivariate logistic regression analyses identified a panel of WBCs, CD4^+^ T cell, and D-dimer values that were correlated with good diagnostic accuracy for discriminating mild/moderate patients from severe/critical patients with an area under the curve of 0.962.

**Conclusion:**

Some key baseline laboratory indicators change with disease development. A panel was identified for discriminating mild/moderate patients from severe/critical patients, suggesting that the panel could serve as a potential biomarker to enable physicians to provide timely medical services in clinical practice.

## Introduction

The Omicron variant shows high infectivity and antibody evasion. It was first discovered in South Africa and Botswana in November 2021 and accounted for 34% of all SARS-CoV-2 strains by 28 May 2022 (1). Although the severity rate and death rate associated with Omicron infection are low, more than 9,000 people in Hong Kong have lost their lives to this variant (2).

During the global Omicron pandemic, patients aged 60 years or above were regarded as having a poor prognosis according to national COVID-19 guidance (3). Over 95% of patients who lost their lives in Hong Kong were older adults over the age of 60 years (2). Shanghai has been amid the Omicron pandemic since March 2022, and by July 2022, more than six hundred thousand infections had been documented (4). Few studies have explored the risk factors for severe/critical Omicron variant BA.2 infections among older adults; understanding these risk factors would promote improvement in the public health system and reduction in mortality. Therefore, to evaluate risk factors for severe/critical Omicron infection in older adults, we conducted a study of seniors during the Shanghai Omicron pandemic.

A decrease in lymphocytes is the most common characteristic of COVID-19 infection. Moreover, lymphocytopenia is associated with severe COVID-19 infection and is correlated with poor prognosis (5–7). Research has demonstrated that booster doses of the inactive vaccine BBIBP-CORV have better protection against SARS-CoV-2-related variants (8). Ratios of TNF-α-producing CD4^+^ T lymphocytes were higher in patients vaccinated with inactive vaccines following SARS-CoV-2 antigen stimulation (9). Another study suggested that an inactivated virus vaccine can elicit a T-lymphocyte response, and its mechanism is to target not only anti-spike proteins but also the nucleocapsid and membrane (10, 11). CD4^+^ T lymphocytes are key in mediating protective immunity by producing virus-specific antibodies. CD8^+^ T lymphocytes clear infected cells and control different types of viruses by secreting cytokines (12).

However, whether some key baseline laboratory indicators can be potential biomarkers for disease progression has not been well explored. Our study aimed to explore the risk factors as a potential biomarker for differentiating a mild/moderate state from a severe/critical state in patients with Omicron infection.

## Materials and methods

### Study design and patient enrollment

This study was conducted at Shanghai Ninth People’s Hospital from April 2022 to May 2022. All infections were confirmed in patients aged 60 years or above with SARS-CoV-2 real-time polymerase chain reaction (RT-PCR). The Ethics Committee of Shanghai Ninth People’s Hospital approved this study (number: SH9H-2022-T123-2).

The diagnostic criteria and management recommendations were conducted following the National COVID-19 9th edition guidelines (13). Mild Omicron infection was defined as follows: mild clinical symptoms and no pneumonia manifestation on imaging. Moderate Omicron infection was defined as follows: symptomatic and typical ground-glass opacity lesions on lung imaging. Severe Omicron infection was determined by any of the following: respiratory rate of ≥30 breaths/min, oxygen saturation of ≤93% at rest, arterial partial pressure of oxygen (PaO2)/oxygen concentration (FiO2) of ≤300 mmHg, and >50% of lesion progression within 24–48 h. At least one of the following diagnostic criteria of critical infections was included: (1) shock, (2) intensive care unit management required because of a combination of other organ failures, or (3) respiratory failure requiring ventilator treatment. Patients younger than 60 years, without data for lymphocyte subsets or with an unclear medical history, were excluded.

Participants received SARS-CoV-2 RT-PCR tests using the SARS-CoV-2 ZC-HX-201-2 kit (Biogerm, Shanghai, China) during hospitalization once a day. When two continuous negative SARS-CoV-2 RT-PCR tests were measured (the cycle threshold values of both the N gene and ORF1ab gene were >35), viral clearance was considered. Serum lymphocyte subset counts, including CD4^+^ T cells, CD8^+^ T cells, B cells, and NK cells, were measured by flow cytometry, which is a powerful tool for quantitative analysis and cell sorting. Routine blood and coagulation function data were also collected.

**FIGURE 1:**
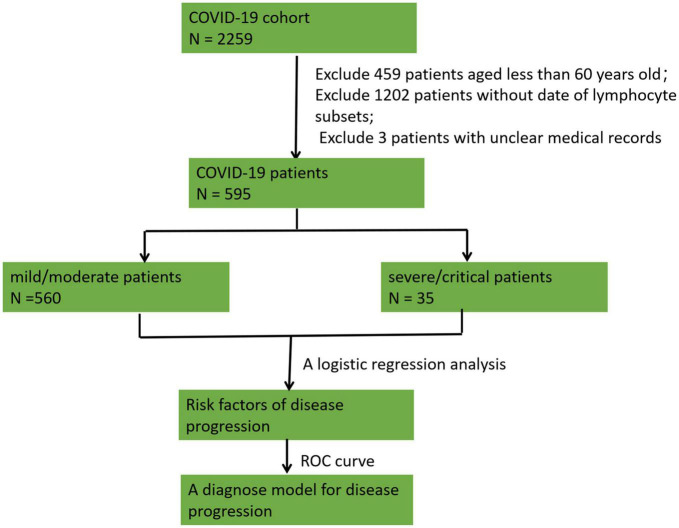
A flow chart of patient enrollment and exclusion and the study design. A total of 2,259 patients with COVID-19 infection were considered. We excluded 459 patients aged <60 years, 1,202 patients without lymphocyte subset data, and 3 participants with unclear medical records. Overall, 595 patients were enrolled in the study. The patients were divided into a mild/moderate group and a severe/critical group, and a logistic regression analysis was conducted to analyze risk factors for disease progression. Receiver operating characteristic (ROC) curves were constructed to explore the ability to discriminate severe/critical patients from mild/moderate patients.

The baseline characteristics, including epidemiologic information, comorbidities, clinical manifestations, laboratory examinations, and prognosis, were collected. The patients were divided into a mild/moderate group and a severe/critical group following the national COVID-19 9th edition guidelines. Risk factors were analyzed to discriminate severe/critical patients from mild/moderate patients among geriatric individuals by logistic regression. Receiver operating characteristic (ROC) curves were generated to explore the ability to discriminate severe/critical patients from mild/moderate patients. A flow chart of patient enrollment and exclusion and the study design is shown in Figure 1.

### Statistical analyses

Continuous variables are expressed as the mean values ± standard deviations, and the Kolmogorov?Smirnov test was conducted for normally distributed data; otherwise, variables are described by the median and interquartile range (IQR). Pearson’s chi-square test was conducted to evaluate two independent binomial variables. The risk factors for severe/critical patients were analyzed by logistic regression, and odds ratios (ORs) and 95% confidence intervals (CIs) were calculated. *P* < 0.05 was considered to be statistically significant. ROC curves were constructed to explore the ability to discriminate severe/critical patients from mild/moderate patients. G*Power 3.1.9.2 was used to calculate the sample number. SPSS (v23.0) software was used for statistical analysis, and GraphPad Prism (v 8) was used to generate figures.

## Results

### Baseline characteristics of geriatric Omicron infections

A total of 2,259 patients with COVID-19 infection were included; of which, we excluded 459 patients aged <60 years, 1,202 patients without lymphocyte subset data, and 3 participants with unclear medical records. Overall, 595 patients were enrolled in the study. The basic patient features and laboratory tests are listed in Table 1. The median age of the study subjects was 76 years (IQR: 69–85), and the percentages of participants aged 61–70, 71–80, and over 80 years were 31.6% (188/595), 33.6% (200/595), and 34.8% (207/595), respectively. Most of the eligible patients (69.1%, 411/595) were unvaccinated, followed by patients who had received a two-dose vaccination (14.1%, 84/595), and a third booster dose vaccination (16.0%, 95/595).

**TABLE 1:** The basic characteristics of the enrolled patients.

*N*	595
Age, median (IQR)	75 (69–85)
61–70 years (*N*, %)	188, 31.6%
71–80 years (*N*, %)	200, 33.6%
>80 years (*N*, %)	207, 34.8%
**Gender, *N* (%)**	
Male	292, 49.1%
Female	303, 50.9%
**Vaccination, *N* (%)**	
Unvaccinated	411, 69.1%
One-dose	5, 0.8%
Two doses	84, 14.1%
Three doses	95, 16.0%
Symptoms on admission, *N* (%)	345, 58.0%
Viral pneumonia on CT, *N* (%)	344, 57.8%
**Clinical diagnosis**	
Mild	251, 42.2%
Moderate	309, 51.9%
Severe	18, 3.0%
Critical	17, 2.9%
**Symptoms, *N* (%)**	
Fever	98 16.5%
Sore throat	109, 18.3%
Cough	267, 44.9%
Diarrhea	1, 0.2%
Nasal obstruction or rhinorrhea	49, 8.2%
Impaired sense of smell	1, 0.2%
**Treatment, *N* (%)**	
Respiratory support	108, 18.2%
Dialysis	3, 0.5%
Antiviral drug	323, 54.3%
Glucocorticoid	77, 12.9%
Viral shedding time, median (IQR)	9 (6–12)
**Outcome, *N* (%)**	
Death	5, 0.8%
Relieve	590, 99.2%
**Laboratory examinations**	
WBCs < 3.5 ×10^9/L	26/595, 4.4%
Lymphocytes < 1 ×10^9/L	92/595, 15.5%
ALT > 50 U/L	54/588, 9.2%
AST > 50 U/L	49/588, 8.3%
D-dimer > 0.5 mg/L	309/590, 52.4%
CRP > 10 mg/L	163/595, 27.4%
IL1β > 5 pg/mL	127/579, 22.0%
IL2R > 710 U/mL	140/490, 28.6%
IL6 > 3.4 pg/mL	479/577, 83.0%
IL10 > 9.1 pg/mL	38/490, 7.8%
LDH > 250 U/L	162/530, 30.6%
BNP > 100 pg/mL	147/547, 26.9%

WBCs, white blood cells; ALT, alanine aminotransferase; AST, aspartate transaminase; CRP, C-reactive protein; IL, interleukin; LDH, lactate dehydrogenase; BNP, N-terminal pro-B-type natriuretic peptide.

According to the national COVID-19 guidelines (version 9), the enrolled patients were classified into four clinical groups, namely, mild (42.2%, 251/595), moderate (51.9%, 309/595), severe (3.0%, 18/595), and critical (2.9%, 17/595). Baseline laboratory tests at the time of hospitalization were analyzed. Lymphocyte counts were less than 1 × 10^9/L in 15.5% (92/595) of patients. D-dimer and interleukin 6 (IL-6) were elevated in more than half of the patients. The abnormal incidence rates of N-terminal pro-B-type natriuretic peptide (BNP) and lactate dehydrogenase (LDH) were 26.9 and 26.9%, respectively. A total of 99.2% (590/595) of patients were discharged, and 5 (0.8%) patients died.

### Differential laboratory examinations between patients with severe and non-severe COVID-19 infection

**FIGURE 2:**
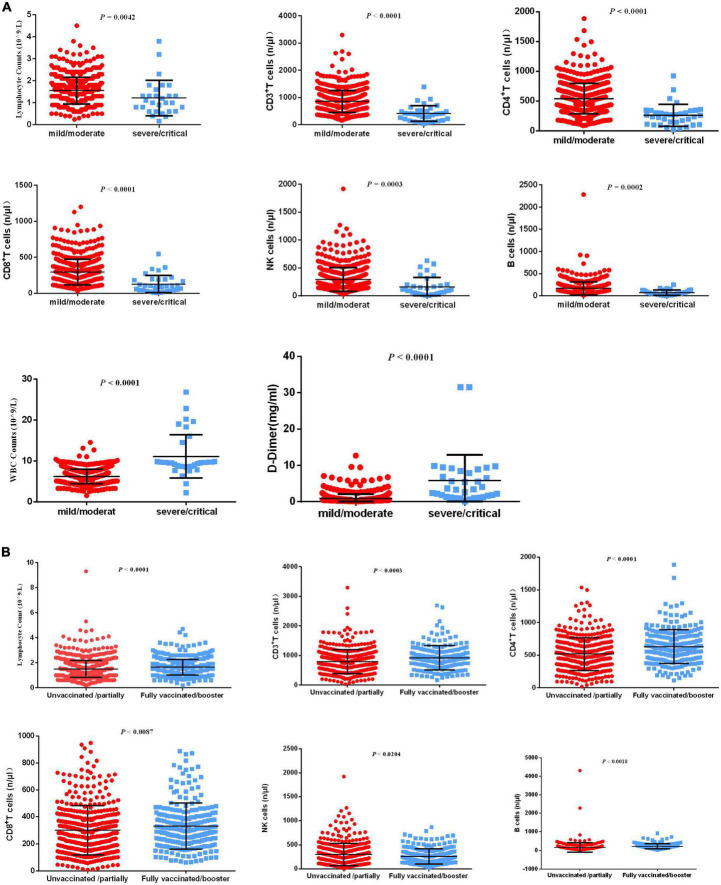
Differential laboratory examinations of patients with COVID-19 infection. **(A)** The indicators including lymphocyte counts, CD3^+^ T cells, CD4^+^ T cells, CD8^+^ T cells, NK cells, B cells, WBCs, and D-dimer levels were analyzed between patients with severe/critical COVID-19 infection and patients with mild/moderate COVID-19 infection. **(B)** The indicators, including lymphocyte counts, CD3^+^ T cells, CD4^+^ T cells, CD8^+^ T cells, NK cells, and B cells, were analyzed between unvaccinated/partially vaccinated patients and fully vaccinated/booster-vaccinated patients.

Decreased lymphocytes are the most common characteristic of COVID-19 infections. Recent research demonstrated that lymphocytopenia is associated with severe COVID-19 infection and is correlated with poor clinical prognosis (5–7). The levels of lymphocyte subsets are not fully clear among older adults. The pathological changes in the lung make it prone to pulmonary vasculitis and thrombosis (14), and systemic microvascular thrombosis may occur in most patients who die (15). However, less is known about D-dimer levels in the progression of COVID-19. Decreased absolute neutrophil or leukocyte counts at the early stages of COVID-19 infection have been widely revealed (16), and the level of white blood cells (WBCs) has not been fully evaluated during the progression of COVID-19. Therefore, we evaluated the levels of serum lymphocyte subsets, D-dimer, and WBCs from patients with severe and non-severe COVID-19 infection. The results demonstrated that lymphocyte counts and levels of CD3^+^ T cells, CD4^+^ T cells, CD8^+^ T cells, NK cells, and B cells were significantly decreased, and WBCs and D-dimer were significantly increased in patients with severe/critical COVID-19 infection compared to patients with mild/moderate COVID-19 infection (Figure 2A).

Although neutralizing activity was reduced, inactivated vaccination still produced a better response from memory cells (8). T-cell responses alleviated disease progression caused by COVID-19 variants (17). The levels of lymphocyte subsets have not been fully evaluated in unvaccinated/partially vaccinated geriatric individuals or fully vaccinated/booster-vaccinated geriatric individuals. Therefore, we evaluated serum lymphocyte subsets in unvaccinated/partially vaccinated and fully vaccinated/booster-vaccinated geriatric individuals. The results demonstrated that lymphocyte counts and levels of CD3^+^ T cells, CD4^+^ T cells, CD8^+^ T cells, NK cells, and B cells were highly increased in fully vaccinated/booster-vaccinated patients compared with unvaccinated/partially vaccinated patients (Figure 2B).

### Risk factors for disease progression to a severe/critical state

Previous research demonstrated that compared with a placebo, nirmatrelvir plus ritonavir reduced the risk of developing severe COVID-19 disease (18). A significant increase in mortality was associated with previous stroke and chronic kidney disease in elderly inpatients (9). In our study, the risk factors for disease progression to a severe/critical state were analyzed by univariate and multivariate logistic regression analyses. Univariate regression analysis demonstrated that >80 years old, unvaccinated/partially vaccinated, increased WBCs and D-dimer levels, decreased lymphocyte counts and levels of CD3^+^ T cells, CD4^+^ T cells, CD8^+^ T cells, B cells, and NK cells, chronic kidney disease, and cerebrovascular disease were hazard factors for disease progression (Table 2). Using multivariate regression analysis, we found that patients with increased WBCs and D-dimer levels and decreased CD4^+^ T cells were more likely to progress to a severe/critical state (adjusted OR of WBCs, 1.726 [95% CI: 1.388–2.147], *P* < 0.0001; adjusted OR of D-dimer 1.329 [95% CI: 1.126–1.568], *P* < 0.0001; and adjusted OR of CD4^+^ T cells 0.993 [95% CI: 0.990–0.997], *P* < 0.0001). The predicted probability of the risk of disease progression to a severe/critical state from the logit model was based on the panel Logit (P) = −5.738 + 0.586 × WBCs-0.006 × CD4^+^ T cells + 0.306 × D-dimer.

**TABLE 2:** Risk factors for progressing to severe/critical disease among geriatric patients.

	Univariate		Multivariate	
	OR	*P*	Adjusted OR	*P*
Female	0.623 (0.310–1.250)	0.183	/	/
Age groups (years)				
<70	1		/	/
70–80	2.153 (0.734–6.318)	0.163	/	/
>80	3.660 (1.339–10.001)	0.011	/	/
Vaccination status				
Unvaccinated/Partially vaccinated	1		/	/
Fully vaccinated/Booster	0.131 (0.031–0.553)	0.006	/	/
WBCs	1.975 (1.588–2.457)	0.000	1.726 (1.388–2.147)	0.000
Lymphocytes	0.332 (0.156–0.704)	0.004	/	/
CD3	0.995 (0.993–0.997)	0.000		
CD4	0.993 (0.990–0.995)	0.000	0.993 (0.990–0.997)	0.000
CD8	0.988 (0.983–0.992)	0.000	/	/
NK	0.994 (0.990–0.997)	0.000	/	/
B	0.982 (0.975–0.990)	0.000	/	/
D-dimer	1.738 (1.502–2.011)	0.000	1.329 (1.126–1.568)	0.000
Cerebrovascular disease	0.369 (0.170–0.800)	0.012	/	/
Chronic kidney disease	0.245 (0.094–0.640)	0.004	/	/

### The panel can discriminate the severe/critical state from the mild/moderate state

**FIGURE 3:**
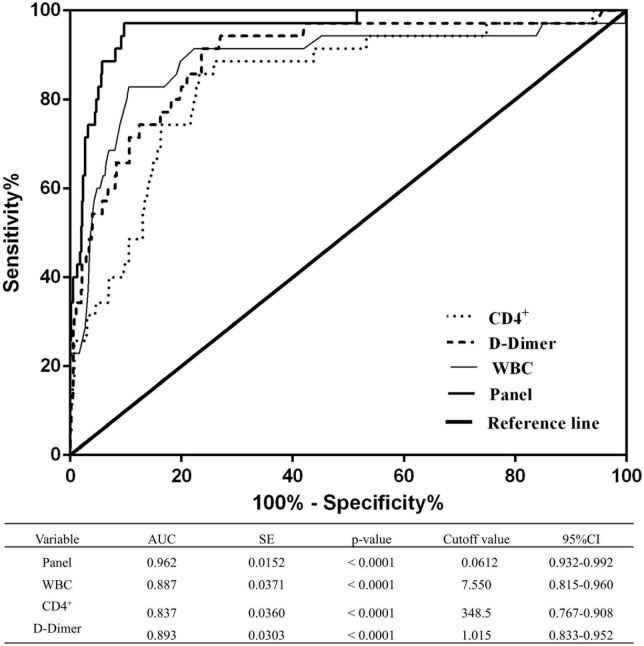
Receiver operating characteristic curves of WBCs, CD4^+^ T cells, D-dimer levels, and the combined panel for distinguishing the severe/critical state from the mild/moderate state. An analysis of the ROC curves for WBCs, CD4^+^ T cells, D-dimer levels, and the combined panel was used to differentiate the severe/critical state from the mild/moderate state. Areas under the curves were compared. SE, standard error; CI, confidence interval.

The diagnostic performance of the established panel of the risk of disease progression to a severe/critical state was conducted by ROC analysis. The AUC was 0.962 with specificity = 90.27% and sensitivity = 97.14% (Figure 3), which demonstrated that the panel has a high specificity and sensitivity for discriminating severe/critical state from a mild/moderate state.

## Discussion

Early identification and diagnosis of geriatric patients with a severe/critical Omicron infection vs. a mild/moderate infection enable physicians to provide timely medical services in clinical practice. Hence, it is necessary to identify risk factors for early identification and diagnosis. This research revealed the key baseline characteristics, changes in levels of lymphocyte subsets, WBCs and D-dimer with disease progression, and a panel for discriminating mild/moderate patients from severe/critical patients.

Research has demonstrated that booster doses of the inactive vaccine BBIBP-CORV have better protection against SARS-CoV-2-related variants (8), and virus-specific antibodies were detected in 91.48% of vaccine participants on Day 28 after the second vaccine dose (19). Although neutralizing activity is reduced with inactivated vaccination, it still produced a better response from memory cells (8). T-cell responses alleviated disease progression caused by COVID-19 variants that partially or largely evade neutralizing antibodies, which is in good agreement with T-cell-mediated immunity in humans for influenza (17). Our results demonstrated that lymphocyte counts and levels of CD3^+^ T cells, CD4^+^ T cells, CD8^+^ T cells, B cells, and NK cells were highly increased in fully vaccinated/booster-vaccinated older adult patients compared with unvaccinated/partially vaccinated older adult patients, which is consistent with a previous study (20–22).

CD4^+^ T lymphocytes are key in mediating protective immunity by producing virus-specific antibodies. CD8^+^ T lymphocytes clear infected cells and control different types of viruses by secreting cytokines. Research has demonstrated that the decrease in total T cells, CD4^+^ T cells, and CD8^+^ T cells is negatively correlated with patient survival, and T cells from patients with COVID-19 infection highly increase the exhaustion marker PD-1 (23–25). This study revealed that lymphocyte counts and levels of CD3^+^ T cells, CD4^+^ T cells, CD8^+^ T cells, B cells, and NK cells were significantly decreased in patients with severe/critical COVID-19 infection compared to patients with mild/moderate COVID-19 infection, which was consistent with a previous study (23, 26, 27). These results suggest T-cell dysfunction and immune exhaustion during COVID-19 infection in older adults.

Compared with a placebo, nirmatrelvir plus ritonavir reduces the risk of developing severe COVID-19 infection (18). The monoclonal antibody was correlated with a significantly downregulated SARS-CoV-2 viral load (28). Significant mortality was increased from previous stroke and chronic kidney disease in elderly inpatients (9). Over 95% of patients who died were older adults aged 60 years or above in Hong Kong during the Omicron wave (2). Therefore, univariate and multivariate logistic regression analyses were conducted to analyze the key indicators. Univariate regression analysis demonstrated that being >80 years old, being unvaccinated/partially vaccinated, and having increased WBCs and D-dimer levels, decreased lymphocyte counts and levels of CD3^+^ T cells, CD4^+^ T cells, CD8^+^ T cells, B cells, and NK cells, chronic kidney disease, and cerebrovascular disease were risk factors for COVID-19 progression. Furthermore, multivariate regression analysis indicated that increased WBC and D-dimer levels and decreased CD4^+^ T cell levels were more likely to progress to a severe/critical state; the AUC was 0.962 with 90.27% of specificity and 97.14% of sensitivity, which demonstrated that the panel has high sensitivity and specificity for discriminating a severe/critical state from a mild/moderate state.

Decreased absolute neutrophil or leukocyte counts at the early stages of COVID-19 infection have been widely revealed (16) and are considered a consequence of either peripheral damage or bone marrow suppression in the early stages of infection (29). Moreover, our study demonstrated that leukocyte counts were significantly increased in the severe group with COVID-19 progression, which is consistent with previous retrospective studies (30) and other meta-analyses (31). The possible reason was that the induction of circulating leukocytes and neutrophils was commonly upregulated following the viral invasion. This may increase the vulnerability of secondary bacterial infections in patients with severe COVID-19 infections, which are characterized by leukocytosis (32). Furthermore, compared with patients with non-severe COVID-19 infection, patients with severe/critical COVID-19 infection showed steadily upregulated leukocytosis and neutrophilia in dynamic laboratory results, which is considered to be a consequence of the secondary infection and cytokine storm caused by the infection (30, 33).

When a person is infected with COVID-19 through the respiratory tract or close contact, pathological changes in the lung make it prone to pulmonary vasculitis and thrombosis (14), and systemic microvascular thrombosis can occur in the main small vessels of the whole body (15, 34). Therefore, anticoagulants may have certain benefits for patients with severe COVID-19 infection, especially those without cardiovascular disease (35). The D-dimer level is extremely important in the diagnosis, efficacy evaluation, and prognosis of thrombotic diseases. Previous research demonstrated that baseline-increased D-dimer was correlated with a poor prognosis of COVID-19 (36). Dynamic changes in the D-dimer level are significantly associated with the prognosis of patients with COVID-19, and a D-dimer level of >11 g/ml is associated with a decreased mortality rate after heparin treatment (37). Our study revealed that D-dimer levels were highly upregulated in severe/critical patients compared to that in mild/moderate patients, suggesting that timely and effective anticoagulant treatment may be effective.

There are some limitations to this study worth mentioning. First, we recruited only some older adults in Shanghai, and most of them had stable chronic medical conditions. To obtain more comprehensive data, we should include more centers and participants with different health statuses. Second, there is a lack of data on laboratory tests, which can lead to bias in data interpretation.

In conclusion, some key baseline characteristics, especially levels of CD4^+^ T cells, D-dimer, and WBCs, changed with disease development. A panel was identified that was able to discriminate mild/moderate patients from severe/critical patients, suggesting that the panel could serve as a potential biomarker to enable physicians to provide timely medical services in clinical practice.

## Data availability statement

The original contributions presented in this study are included in the article/supplementary material, further inquiries can be directed to the corresponding authors.

## Ethics statement

The studies involving human participants were reviewed and approved by the Shanghai Ninth People’s Hospital, Shanghai Jiao Tong University School of Medicine. The Ethics Committee waived the requirement of written informed consent for participation. Written informed consent was not obtained from the individual(s) for the publication of any potentially identifiable images or data included in this article.

## Author contributions

SB, XY, and JX contributed to the research design and statistical analysis. SB, GL, XY, and JX helped in the analysis. SB contributed to the manuscript writing and funding. XY and JX contributed to the critical revision and helped with the administration and technical support. All authors contributed to the data acquisition.
